# Supplementary material for: Islet autoantibodies as precision diagnostic tools to characterize heterogeneity in type 1 diabetes: a systematic review
Source: Commun Med (Lond). 2024 Apr 6;4:66. doi: 10.1038/s43856-024-00478-y (PMC10998887; doi:10.1038/s43856-024-00478-y)
Supplement: Supplementary file 1 — Supplementary Information [file 43856_2024_478_MOESM1_ESM.pdf]

Supplementary Note 1. Search strategy.

2022-06-14

Pubmed

#1
("Precision Medicine"[Mesh] OR Subtype*[Title/Abstract] OR heterogeneity[Title/Abstract] OR heterogeneity[Title/Abstract] OR endotype*[Title/Abstract] OR personalized[Title/Abstract] OR tailored[Title/Abstract] OR strat*[Title/Abstract] OR subgroup*[Title/Abstract] OR variability[Title/Abstract] OR phenotype*[Title/Abstract] OR pattern*[Title/Abstract] OR predict*[Title/Abstract] OR stage [Title/Abstract] OR variant*[Title/Abstract] OR predict*[Title/Abstract] OR risk*[Title/Abstract] OR genetic risk score*[Title/Abstract] OR signature*[Title/Abstract] OR ("genetic predisposition to disease"[MeSH Terms]))
=8216498

#2
"Diabetes Mellitus, Type 1 "[Mesh]
=83221

#3
#1 AND #2
=29779

#4
(review*[Title/Abstract]) OR (review*[Publication Type])
=4132084

#5
#3 NOT #4
=23830

#6
#7 Filters: Humans, English, from 2011 – 2022
=9941

Supplementary Table 1. Non-European populations included in review.

Populations from a Non-European Ancestry Studied	Number of papers
Brazilian	2
Chinese	2
Japanese	2
European Caucasians, Moghrabin Caucasians, Black Africans, and Mixed Origin	1
Mexican	1
Tunisian	1
Qatari	1
