# Supplementary material for: Islet autoantibodies as precision diagnostic tools to characterize heterogeneity in type 1 diabetes: a systematic review
Source: Commun Med (Lond). 2024 Apr 6;4:66. doi: 10.1038/s43856-024-00478-y (PMC10998887; doi:10.1038/s43856-024-00478-y)
Supplement: Supplementary file 2 — Description of Supplementary Data Files [file 43856_2024_478_MOESM2_ESM.docx]

**Description of Additional Supplementary Files**

**File Name:** Supplementary Data 1

**Description:** Autoantibody features characterize progression before T1D diagnosis.

**File Name:** Supplementary Data 2

**Description:** Autoantibody features characterize heterogeneity at T1D diagnosis.

**File Name:** Supplementary Data 3

**Description:** Source Data for Figure 2.

**File Name:** Supplementary Data 4

**Description:** Supplementary Abbreviations.

**File Name:** Supplementary Data 5

**Description:** Supplementary References.

**File Name:** Supplementary Data 6

**Description:** Alphabetized list of all identified papers.
